# Proposing new early detection indicators for pancreatic cancer: Combining machine learning and neural networks for serum miRNA-based diagnostic model

**DOI:** 10.3389/fonc.2023.1244578

**Published:** 2023-08-03

**Authors:** Hao Chi, Haiqing Chen, Rui Wang, Jieying Zhang, Lai Jiang, Shengke Zhang, Chenglu Jiang, Jinbang Huang, Xiaomin Quan, Yunfei Liu, Qinhong Zhang, Guanhu Yang

**Affiliations:** ^1^ Clinical Medical College, Southwest Medical University, Luzhou, China; ^2^ Department of General Surgery (Hepatobiliary Surgery), The Affiliated Hospital of Southwest Medical University, Luzhou, China; ^3^ Nuclear Medicine and Molecular Imaging Key Laboratory of Sichuan Province, Luzhou, China; ^4^ Academician (Expert) Workstation of Sichuan Province, Luzhou, China; ^5^ First Teaching Hospital of Tianjin University of Traditional Chinese Medicine, Tianjin, China; ^6^ National Clinical Research Center for Chinese Medicine Acupuncture and Moxibustion, Tianjin, China; ^7^ Beijing University of Chinese Medicine, Beijing, China; ^8^ Beijing University of Chinese Medicine Second Affiliated DongFang Hospital, Beijing, China; ^9^ Department of General, Visceral, and Transplant Surgery, Ludwig-Maximilians-University Munich, Munich, Germany; ^10^ Shenzhen Frontiers in Chinese Medicine Research Co., Ltd., Shenzhen, China; ^11^ Department of Specialty Medicine, Ohio University, Athens, OH, United States

**Keywords:** pancreatic cancer, artificial intelligence, early diagnosis, serum miRNA, machine learning, therapy

## Abstract

**Background:**

Pancreatic cancer (PC) is a lethal malignancy that ranks seventh in terms of global cancer-related mortality. Despite advancements in treatment, the five-year survival rate remains low, emphasizing the urgent need for reliable early detection methods. MicroRNAs (miRNAs), a group of non-coding RNAs involved in critical gene regulatory mechanisms, have garnered significant attention as potential diagnostic and prognostic biomarkers for pancreatic cancer (PC). Their suitability stems from their accessibility and stability in blood, making them particularly appealing for clinical applications.

**Methods:**

In this study, we analyzed serum miRNA expression profiles from three independent PC datasets obtained from the Gene Expression Omnibus (GEO) database. To identify serum miRNAs associated with PC incidence, we employed three machine learning algorithms: Support Vector Machine-Recursive Feature Elimination (SVM-RFE), Least Absolute Shrinkage and Selection Operator (LASSO), and Random Forest. We developed an artificial neural network model to assess the accuracy of the identified PC-related serum miRNAs (PCRSMs) and create a nomogram. These findings were further validated through qPCR experiments. Additionally, patient samples with PC were classified using the consensus clustering method.

**Results:**

Our analysis revealed three PCRSMs, namely hsa-miR-4648, hsa-miR-125b-1-3p, and hsa-miR-3201, using the three machine learning algorithms. The artificial neural network model demonstrated high accuracy in distinguishing between normal and pancreatic cancer samples, with verification and training groups exhibiting AUC values of 0.935 and 0.926, respectively. We also utilized the consensus clustering method to classify PC samples into two optimal subtypes. Furthermore, our investigation into the expression of PCRSMs unveiled a significant negative correlation between the expression of hsa-miR-125b-1-3p and age.

**Conclusion:**

Our study introduces a novel artificial neural network model for early diagnosis of pancreatic cancer, carrying significant clinical implications. Furthermore, our findings provide valuable insights into the pathogenesis of pancreatic cancer and offer potential avenues for drug screening, personalized treatment, and immunotherapy against this lethal disease.

## Introduction

Pancreatic cancer (PC) is an extremely lethal malignancy and holds the unfortunate position of being the seventh leading cause of cancer-related deaths worldwide (1). Alarmingly, the incidence of PC continues to rise, with nearly 500,000 new cases being reported each year (2). Among the various subtypes, pancreatic ductal adenocarcinoma stands out as the most prevalent, presenting an abysmal five-year survival rate of merely 6% (3, 4). This aggressive cancer typically originates from the exocrine duct epithelial cells of the pancreas, progressing through a complex series of non-invasive precursor lesions (5). Several well-established risk factors for PC have been identified, including smoking, alcohol consumption, obesity, diabetes, pancreatic steatosis, and hypercholesterolemia (6–9). Pancreatic cancer primarily manifests in the pancreatic head, accounting for approximately 70% of cases, and it often exhibits a lack of specific clinical symptoms, further complicating early detection and diagnosis. While some common symptoms such as obstructive jaundice, dark urine, loss of appetite, fatigue, and exocrine pancreatic insufficiency may occur, they are not exclusive to PC and can be present in other conditions as well (10–12). Moreover, severe cachexia frequently accompanies pancreatic cancer, contributing significantly to cancer-related mortality. Consequently, relying solely on clinical symptoms for the diagnosis of pancreatic cancer is unreliable and insufficient (13). Currently, techniques such as endoscopic ultrasound-guided fine-needle aspiration (EUS-FNA) or surgical pathology with frozen section analysis (SPACE) are employed for the early diagnosis of suspected pancreatic cancer. However, the accuracy of cytological analysis on collected samples can be compromised due to challenges in sampling, the presence of inflammation, or other confounding factors (14, 15). It is noteworthy that pancreatic peri-fat necrosis, a condition often associated with chronic or acute pancreatitis, can mimic the clinical and imaging features of pancreatic cancer, leading to misdiagnosis. In such cases, surgical pathology or percutaneous biopsy is necessary to confirm the presence of malignancy. Additionally, the aspiration of pancreatic fluid carries a high risk of pancreatic fistula formation, further highlighting the limitations and drawbacks of invasive diagnostic procedures (16, 17). Given these challenges, there is an urgent and compelling need for the development of a non-invasive diagnostic method that combines high accuracy and precision in detecting pancreatic cancer. Such an innovative approach would revolutionize the field by enabling early detection and prompt intervention, ultimately improving patient outcomes and survival rates. In light of the current clinical landscape and the limitations of existing diagnostic techniques, our research endeavors to fill this crucial gap by introducing a novel, non-invasive diagnostic modality that promises to revolutionize the early detection and diagnosis of pancreatic cancer.

MiRNAs are short non-coding RNA molecules, typically consisting of 18-25 nucleotides. They are integral players in the intricate landscape of gene regulation, exerting their influence by specifically binding to the 3’-untranslated region (3’-UTR) of target mRNA molecules. Dysregulation and aberrant processing of miRNAs have been implicated in the development of various cancers (18, 19). MiRNAs participate in a wide range of biological processes, including organismal development, disease progression, immune responses, and modulation of cellular processes like proliferation, differentiation, and apoptosis. They can also influence transcription factors, signaling pathways, and growth factors, thereby exerting their functional effects (20). Circulating miRNAs, known as tumor biomarkers, can be extracted from blood samples and remain stable in the bloodstream. They can be encapsulated in extracellular vesicles and other particles, or bind to high-density lipoproteins, protecting them from degradation by RNA hydrolases. This inherent stability makes circulating miRNAs excellent candidates as biomarkers (21, 22).

The integration of microarray technology and bioinformatics analysis has facilitated high-throughput screening of serum substances in pancreatic cancer patients, aiming to identify novel biomarkers for diagnostic purposes (23–25). However, the diagnostic efficacy of these biomarkers remains unsatisfactory due to their varying specificity and sensitivity. In light of this, artificial neural networks (ANNs) have emerged as powerful tools for predictive analysis of both tumor and non-tumor diseases (26, 27). The utilization of machine learning in the identification of tumor biomarkers has garnered remarkable advantages as another tool (28–30). Through its adaptive nature and proficiency in pattern recognition, machine learning excels in extracting crucial attributes from voluminous multidimensional datasets, thereby amplifying our comprehension of tumor progression mechanisms and propelling the attainment of personalized diagnostic and therapeutic interventions (31–33).Given these capabilities, our study aims to develop a diagnostic model based ANN and machine learning by integrating new feature biomarkers. This model seeks to facilitate the early detection of pancreatic cancer, unravel the underlying mechanisms driving pancreatic cancer pathogenesis in patients, and pave the way for potential therapeutic interventions.

This study aimed to identify potential serum miRNA biomarkers for pancreatic cancer (PC) using machine learning methods and the GEO dataset. The specific objectives included screening for three PC-related serum miRNAs (PCRSMs), establishing a risk score for PC patients using an artificial neural network (ANN) diagnostic model, and investigating the correlation between PCRSMs and clinical characteristics of PC patients. The overall goal was to leverage bioinformatics analysis and machine learning techniques to identify highly specific biomarkers, develop a sensitive diagnostic model for early detection of PC, and gain insights for the development of novel treatment strategies.

## Methods

### Data collection of original data

We obtained three non-coding RNA datasets, namely GSE85589, GSE113486, and GSE59856, from the Gene Expression Omnibus (GEO) database (https://www.ncbi.nlm.nih.gov/geo/). The GSE85589 dataset utilized the GPL19117 [miRNA-4] Affymetrix multispecies miRNA-4 array, while the GSE113486 and GSE59856 datasets utilized the GPL21263 3D Gene Human miRNA V21_1.0.0 array and the GPL18941 3D Gene Human miRNA V20_1.0.0 array, respectively. The GSE85589 dataset consisted of serum miRNA samples from 19 healthy individuals and 88 pancreatic cancer (PC) patients. The GSE113486 dataset included serum miRNA samples from 40 PC patients and 100 controls. For validation purposes, the GSE59856 dataset contained serum miRNAs from 150 healthy individuals and 100 PC patients. All three datasets contained additional clinical characteristics such as age and gender.

### Processing of experimental data and identification of miRNAs with altered expression levels

To preprocess the datasets, we first normalized and log2 transformed the GSE85589 and GSE113486 raw data, which were then merged and used as the training set (34). To calculate the gene expression value for each gene, we averaged the expression level of multiple probes targeting the same gene and removed batch effects using the “sva” R package (35). We utilized the “limma” R package to screen for differentially expressed serum miRNAs in pancreatic cancer (36). We selected candidate miRNAs with |log(FC)|>1 and P<0.05 as the cutoff, and identified them as significantly differentially expressed pancreatic cancer-related serum miRNAs (PCRSMs).

### Machine learning-based feature selection of miRNAs

Three machine learning algorithms, including Support Vector Machine-Recursive Feature Elimination (SVM-RFE), LASSO regression analysis, and Random Forest, were employed to select PCRSMs from the candidate miRNAs (37). SVM-RFE, an algorithm rooted in the principle of maximum margin within support vector machines (SVM), facilitates a meticulous backward selection process. It commences by assigning a performance-based score to each feature using the training samples. Subsequently, the feature with the lowest score is systematically eliminated, leading to a retraining of the model using the remaining features. This iterative procedure is executed until the desired number of features is determined (38). LASSO regression analysis embraces the fitting of a generalized linear model while simultaneously undertaking variable selection and complexity adjustment (39). By employing the “glmnet” package with 10-fold cross-validation of penalty parameters, the LASSO regression analysis proficiently determines the most relevant features (40, 41). In addition, the Random Forest algorithm is leveraged to assess the importance of pancreatic cancer-related serum miRNAs and estimate their predictive performance. This algorithm effectively ranks the significance of the miRNAs by employing ten-fold cross-validation. Specifically, serum miRNAs exhibiting a relative importance surpassing the threshold of 1 are regarded as pertinent feature miRNAs (42).

### Artificial neural networks and creating nomograms

The Artificial Neural Network (ANN), an innovative computational model inspired by biological neural networks, exhibits a complex structure comprising interconnected neurons or processing units. ANNs demonstrate a remarkable ability to accurately fit training data and uncover intricate nonlinear relationships among predictor variables. This capacity renders ANNs invaluable for predicting outcomes that have not been previously observed (43). We will use the “neuralnet” R package to establish an artificial neural network model to score disease-specific miRNAs based on the weights of nodes, which can differentiate sample attributes and distinguish whether the sample is diseased (44).To prevent overfitting, regularization techniques such as dropout or L1/L2 regularization may be employed. Furthermore, the dataset is divided into training, validation, and test sets, with the validation set used for hyperparameter tuning and model selection. Performance evaluation metrics, including accuracy, precision, recall, and area under the receiver operating characteristic curve (AUC-ROC), are employed to assess the model’s predictive capacity and generalization ability. At the same time, we will conduct a comprehensive quantitative evaluation of PCRSMs in the form of a nomogram. The calibration curve is a well-established method utilized to evaluate the performance and accuracy of the nomogram. On the other hand, decision curve analysis (DCA) serves as a valuable tool to assess the overall clinical utility and net benefit of the nomogram.

### Consensus clustering

Consensus clustering is a widely used unsupervised method for subtype classification in cancer patients. This approach involves multiple iterations of an inner clustering algorithm, and the results are combined to produce a final consensus clustering solution (45). We employed the “ConsensusClusterPlus” R package to partition the pancreatic cancer patients in our study into distinct ICI clusters, both in the training and validation sets. To determine the optimal number of clusters, we comprehensively evaluated the consensus matrix, the consensus cumulative distribution function (CDF), the CDF curve area, and the tracking plot. Furthermore, we assessed the stability of the clustering solution by performing principal component analysis (PCA) (46).

### qPCR assay

The human pancreatic ductal epithelial cell line, hTERT-HPNE, was obtained from the BeNa Culture Collection in Henan, China. Similarly, the human pancreatic cancer cell lines, PANC-1 and SW1990, were acquired from the Experimental Medicine Center affiliated with the Southwest Medical University in Luzhou, China. The hTERT-HPNE cell line was maintained under standard conditions, with RPMI1640 medium supplemented with 10% fetal bovine serum and 1% penicillin-streptomycin used for cell culture. The hTERT-HPNE cells were cultured under standard conditions at 37°C in a 5% CO2 environment. Likewise, the PANC-1 and SW1990 cells were cultured in DMEM medium supplemented with 10% fetal bovine serum and 1% penicillin-streptomycin, following the same incubation conditions. For RNA extraction, cells in the logarithmic growth phase were harvested, and total RNA was isolated utilizing TRIzol reagent. To assess the distinct expression patterns of miRNAs in hTERT-HPNE, PANC-1, and SW1990 cells, a tailing method combined with quantitative PCR (qPCR) was utilized. The miRNA 1st strand cDNA synthesis kit (Accurate Biotechnology Co., Ltd. Code. AG11717) was utilized to generate the first-strand cDNA of miRNA. Subsequently, the SYBR Green Premix Pro Taq HS qPCR Kit II (Accurate Biotechnology Co., Ltd. Code. AG11702) was employed for quantitative PCR analysis of the reverse transcribed cDNA. Unless otherwise specified, all reagents used in this study were obtained from Gibco (Gibco, Grand Island, NY, USA). The primer sequences utilized in the qPCR assay are listed in **Table 1**.

**Table 1:** The primer sequences for qPCR assay.

Gene	Forward primer	Reverse primer
U6	CTCGCTTCGGCAGCACA	AACGCTTCACGAATTTGCGT
hsa-miR-3201	ATGCGGCCGGGATATGAAGAAAA	_
hsa-miR-125b-1-3p	ACGGGTTAGGCTCTTGGGA	_
hsa-miR-4648	TGTGGGACTGCAAATGGGAG	_

-, represents non-existence. MircoRNAs do not have reverse primers.

### Statistical analysis

Statistical analyses were conducted using the R software (version 4.2.2) and relevant packages. Student’s t-test, Wilcoxon rank-sum test, and Spearman correlation analysis were applied to assess the difference and correlation between variables across different groups. Statistical significance was defined as P<0.05.

## Result

### Identification of candidate PCRSMs

**Figure 1:**
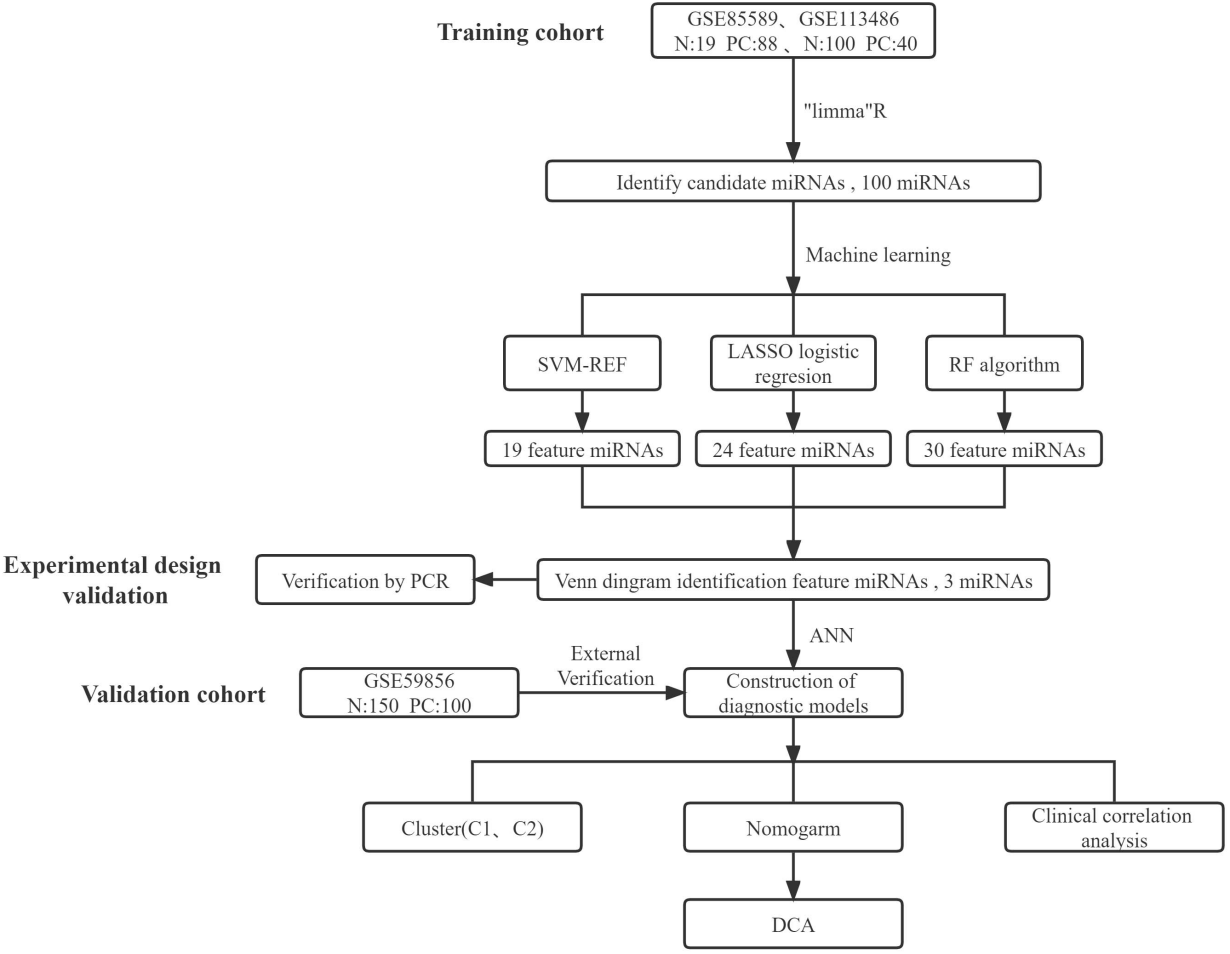
This flowchart summarizes the main design of the study.

**Figure 2:**
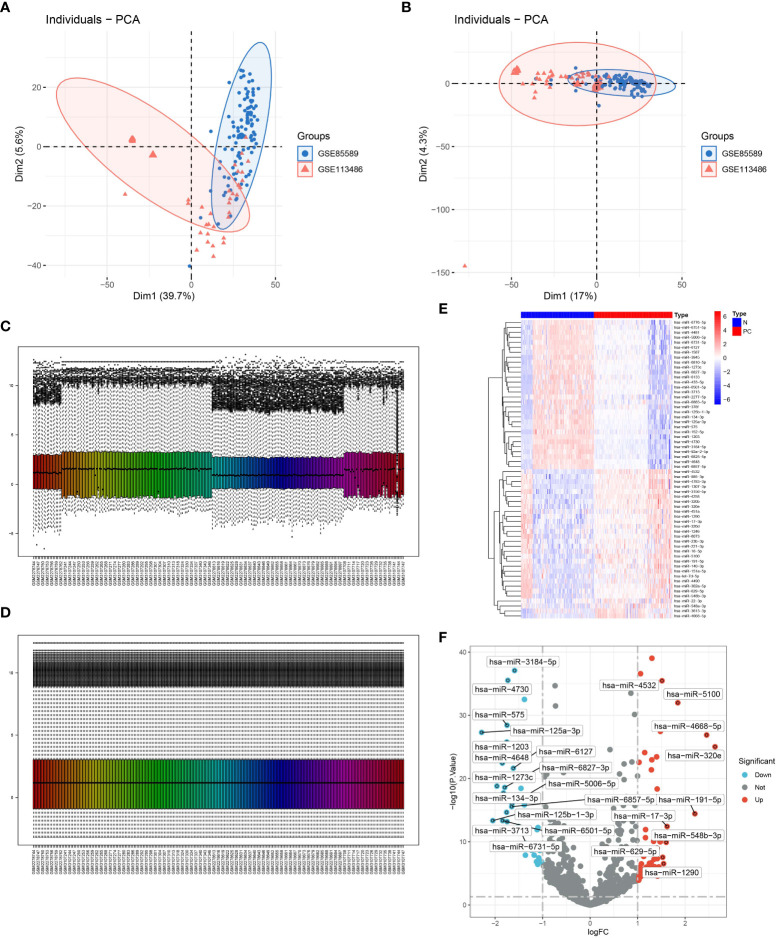
Determination of candidate PCRSMs in GEO expression profiles. **(A, B)** Removal of batch effects. **(C, D)** Visualization of data normalization. **(E, F)** Heatmap and volcano plot showing differentially expressed PCRSMs after merging GSE85589 and GSE113486.

The main study design is depicted in **Figure 1**. Initially, batch correction was performed on the merged data from two GEO series to address batch effects (**Figures 2A, B**). Subsequently, the data were standardized (**Figures 2C, D**). The differential expression analysis of serum miRNAs in pancreatic cancer patients was performed using the “limma” package in the R programming environment. From the analysis, a subset of the top 60 differentially expressed serum miRNAs was identified and chosen for further investigation. A heatmap was generated using the “pheatmap” package in R to visualize the results (**Figure 2E**). Using the criterion of |log2FC|>1, we successfully identified 59 PCRSMs exhibiting up-regulation and 41 PCRSMs showing down-regulation. To visualize the differential expression patterns, a volcano plot was constructed using the “ggplot2” package in the R programming environment (**Figure 2F**).

### Identification of PCRSMs via LASSO regression, support vector machine-recursive feature elimination, and random forest algorithms

**Figure 3:**
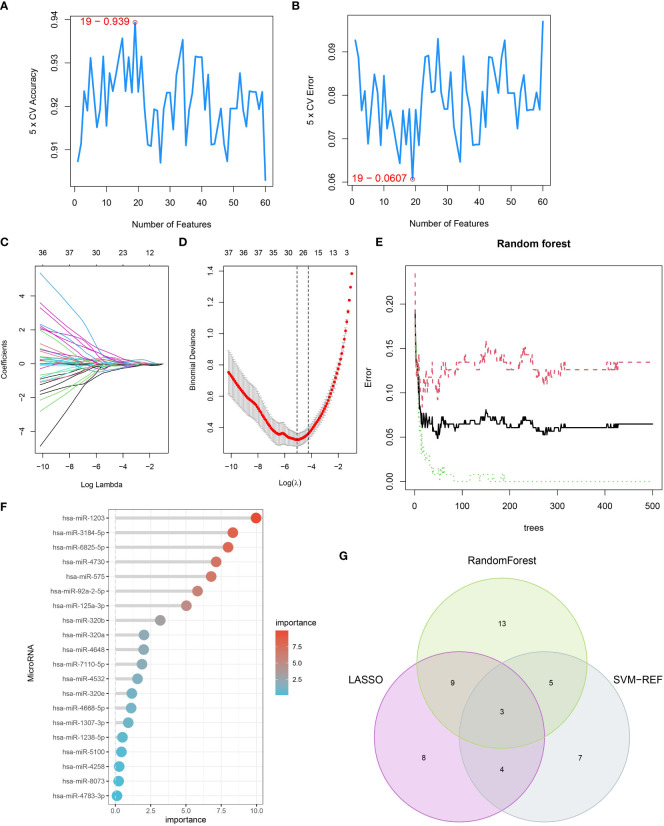
Identification of serum miRNAs associated with pancreatic cancer. **(A, B)** SVM-REF algorithm used for PCRSMs selection, with numbers indicating the optimal selection of 19. **(C)** Ten-fold cross-validation of adjusted parameters selected by the LASSO model, with each line representing an miRNA. **(D)** LASSO coefficient analysis, with the vertical dashed line representing the optimal λ. **(E)** Relationship between the number of trees and error rate in the random forest. **(F)** Relative importance ranking of genes. **(G)** Venn diagram showing the intersection of PCRSMs selected by SVM-REF algorithm, LASSO, and random forest.

To identify potential pancreatic cancer-related serum miRNAs, we employed three machine learning algorithms: LASSO regression, SVM-RFE, and random forest. With SVM-RFE, we selected the top 40 miRNAs, including hsa-miR-3201, hsa-miR-22-3p, hsa-miR-125b-1-3p, hsa-miR-4708-3p, hsa-miR-1307-3p, hsa-miR-2278, hsa-miR-184, hsa-miR-92a-2-5p, hsa-miR-4730, hsa-miR-4648, hsa-miR-27b-3p, hsa-miR-6825-5p, hsa-miR-1246, hsa-miR-1290, hsa-miR-6839-5p, hsa-miR-210-3p, hsa-miR-575, hsa-let-7c-5p, and hsa-miR-221-3p, as feature variables (**Figures 3A, B**). By applying LASSO regression analysis, we identified 24 miRNAs, such as hsa-miR-1307-3p, hsa-miR-4668-5p, hsa-miR-320e, hsa-miR-320a, hsa-miR-7110-5p, hsa-miR-4648, hsa-miR-125b-1-3p, and hsa-miR-3201, as PCRSMs (**Figures 3C, D**). The random forest algorithm showed a stable error rate with approximately 200 decision trees (**Figure 3E**), and 30 miRNAs with relative importance scores greater than 1 were identified as feature variables (**Figure 3F**). Through Venn diagram analysis, we found that three miRNAs, namely hsa-miR-4648, hsa-miR-125b-1-3p, and hsa-miR-3201, were common PCRSMs (**Figure 3G**).

### Diagnostic effects of characteristic PCRSMs

**Figure 4:**
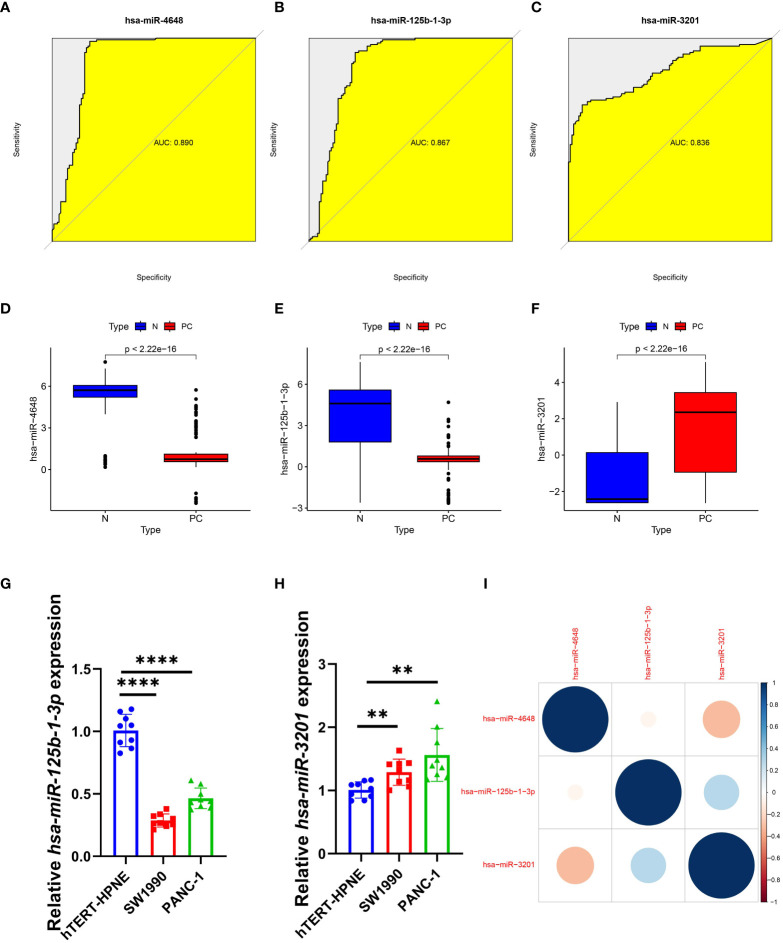
Diagnostic effect and correlation analysis of PCRSMs. **(A–C)** ROC curves for three PCRSMs. **(D–F)** Box plots describing the expression of three PCRSMs in pancreatic cancer and normal control groups. **(G, H)** Verification of three PCRSMs by qRT-PCR. **P < 0.01, ****P < 0.0001. **(I)** Correlation analysis between the three PCRSMs by heatmap.

After conducting an analysis of variance, we evaluated the diagnostic performance of three PCRSMs: hsa-miR-4648, hsa-miR-125b-1-3p, and hsa-miR-3201. The respective ROC curves yielded area under the curve (AUC) values of 0.890, 0.867, and 0.836, demonstrating their potential as diagnostic markers (**Figures 4A–C**). In the merged training dataset, we observed significantly lower expression levels of hsa-miR-4648 and hsa-miR-125b-1-3p in pancreatic cancer patients compared to normal controls (**Figures 4D, E**, P<0.05). Conversely, the expression level of hsa-miR-3201 was higher in the pancreatic cancer group (**Figure 4F**, P<0.05). qRT-PCR analysis confirmed the downregulation of hsa-miR-125b-1-3p and the upregulation of hsa-miR-3201 in pancreatic cancer samples, supporting our initial hypothesis (**Figures 4G, H**, P<0.0001, P<0.01, respectively). Correlation analysis revealed a negative correlation between hsa-miR-4648 and hsa-miR-3201 in the pancreatic cancer group, while hsa-miR-125b-1-3p and hsa-miR-3201 exhibited a positive correlation in the same group (**Figure 4I**). These findings provide insights into the expression patterns and relationships among the selected PCRSMs in pancreatic cancer.

### Establishment of a neural network model and nomogram for predicting pancreatic cancer

**Figure 5:**
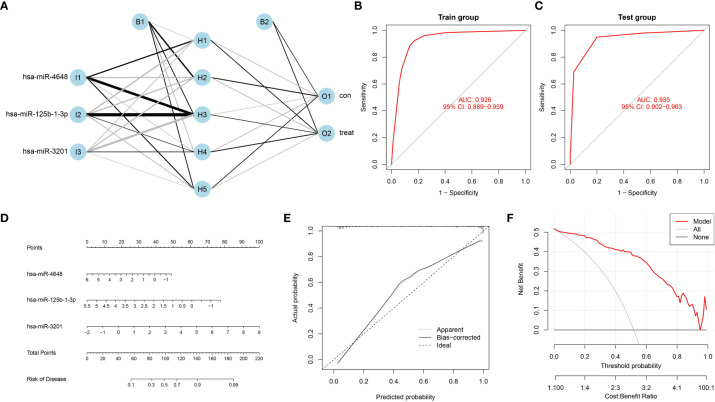
Construction of an artificial neural network model and a bar plot based on PCRSMs. **(A)** Artificial neural network (ANN) model for distinguishing between pancreatic cancer and normal control groups, consisting of three input layers, five hidden layers, and two output layers. **(B, C)** ROC curves of the ANN model diagnostic performance in the training group (GSE85589 and GSE113486 merged) and the validation group (GSE59856). **(D)** Bar plot integrating PC feature miRNAs. **(E)** A calibration curve was constructed to assess the predictive accuracy of the bar plot, providing insights into its reliability and performance. **(F)** Decision curve analysis was conducted to evaluate the clinical utility of the bar plot, demonstrating its potential benefits in guiding clinical decision-making.

To address the need for an efficient and non-invasive diagnostic model for pancreatic cancer, we developed a neural network model with three input layers, five hidden layers, and two output layers. The model incorporated the three characteristic PCRSMs and assigned scores based on their expression levels. A score of 1 was given to up-regulated PCRSMs with expression levels above the median, and 0 otherwise. Conversely, for down-regulated PCRSMs, a score of 1 was assigned for expression levels below the median and 0 otherwise. By categorizing samples into disease and normal groups using assigned scores and weights, the model enabled accurate identification of the disease state (**Figure 5A**). Our model exhibited exceptional performance with remarkable accuracy in both the training and validation ROC curve analyses, as indicated by the AUC values of 0.926 and 0.935, respectively (**Figures 5B, C**). Additionally, we employed the integration of PCRSMs to construct a diagnostic nomogram for pancreatic cancer (**Figure 5D**). Within this nomogram, each PCRSM corresponds to an assigned score, and the cumulative sum of all PCRSM scores determines the total score, which in turn corresponds to distinct risk scores for pancreatic cancer occurrence. The calibration curve showcases the nomogram’s ability to accurately estimate the probability of pancreatic cancer (**Figure 5E**). Moreover, our decision curve analysis underscores the clinical utility of our nomogram, particularly for patients afflicted with pancreatic cancer (**Figure 5F**).

### Identification of serum miRNAs isoforms in pancreatic cancer

**Figure 6:**
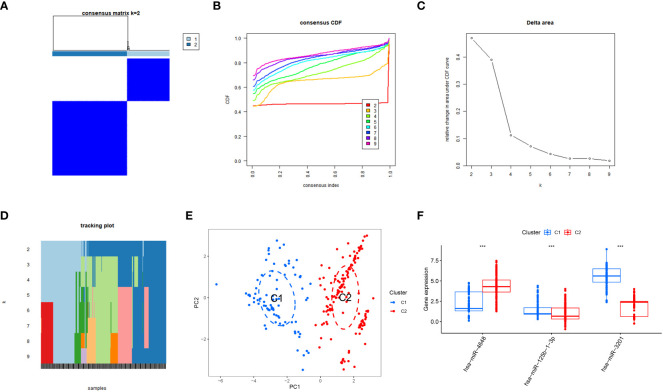
Subtype classification based on PCRSMs. **(A)** Heatmap representing the consensus matrix for K=2, illustrating the clustering patterns. **(B)** Cumulative distribution function of the consensus values for K=2-9, providing insights into the optimal number of subtypes. **(C)** Evaluation of the relative change in the area under the curve (AUC) of the cumulative distribution function. **(D)** Final classification results of the samples for K=2-9, indicating the assigned subtypes. **(E)** Principal Component Analysis (PCA) plot demonstrating the effective segregation of pancreatic cancer patients into two distinct subtypes using PCRSMs. **(F)** Box plots illustrating the differential expression patterns of the PCRSMs between the two identified subtypes. ***, p<0.001

The integration of training and validation sets from GEO datasets was performed to create a unified dataset. Subsequently, a consensus clustering approach was applied to pancreatic cancer (PC) samples, utilizing the expression profiles of three specific PCRSMs. The determination of the optimal number of subtypes, established as 2, was based on the comprehensive analysis of multiple evaluation metrics, including the consensus matrix plot, cumulative distribution function (CDF) plot, relative change in the area under the CDF curve, and tracking plot (**Figures 6A–D**). The two distinct subtypes resulting from the consensus clustering analysis were assigned the labels C1 and C2 to differentiate them, and their distinct nature was confirmed by principal component analysis (PCA), which revealed significant differences between the subtypes (**Figure 6E**). Additionally, the expression boxplot illustrated the differential expression patterns of the two subtypes in the specific PCRSMs (**Figure 6F**). This analysis of subtyping provides valuable information on the heterogeneity of PC, potentially paving the way for more personalized treatment strategies.

### Clinical correlation analysis of pancreatic cancer patients

**Figure 7:**
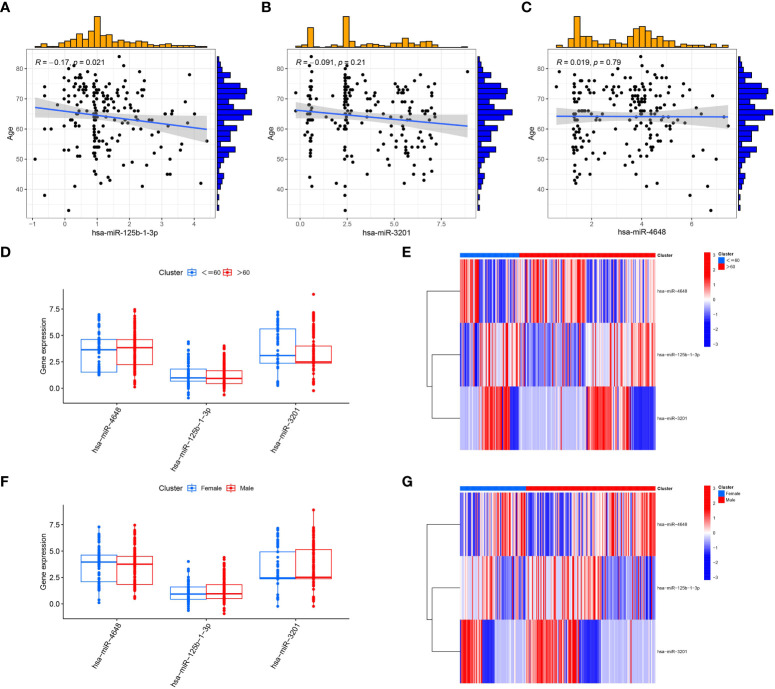
Correlation analysis between PCRSMs and clinical characteristics of pancreatic cancer patients. **(A–C)** Correlation analysis between the expression levels of three PCRSMs and age, with R>0 indicating positive correlation and R<0 indicating negative correlation, and P<0.05 indicating statistical significance. **(D, E)** Box plots and heatmaps showing the expression of PCRSMs in two groups divided by 60 years of age. **(F, G)** Box plots and heatmaps showing the gender differences in the expression of PCRSMs.

In our analysis of pancreatic cancer (PC) patients, we explored the relationship between the expression levels of characteristic PCRSMs and age as well as gender to assess the need for personalized treatment plans. We observed a significant negative correlation between hsa-miR-125b-1-3p and age in PC patients (**Figure 7A**, p<0.05). Although hsa-miR-3201 (**Figure 7B**) and hsa-miR-4648 (**Figure 7C**) showed some correlation with age in PC patients, the associations were not statistically significant (P>0.05). Surprisingly, no significant differences were found in the expression of the three characteristic PCRSMs between PC patients above and below 60 years of age (**Figures 7D, E**), as well as between male and female PC patients (**Figures 7F, G**). These results suggest that gender and age (with 60 as a threshold) may not provide sufficient evidence to warrant personalized treatment plans. However, the downregulation of hsa-miR-125b-1-3p with increasing age could offer a new perspective for targeted drug treatment in PC patients. Subsequent investigations are warranted to validate the present finding and delve deeper into its potential implications.

## Discussion

Pancreatic cancer is a highly invasive malignancy characterized by a global escalation and poor clinical outcomes, including a significantly low five-year survival rate (47, 48). Unfortunately, effective early detection methods for pancreatic cancer are lacking. The current treatment strategies primarily involve surgical resection, chemotherapy, and immunotherapy. Despite notable advancements in pancreatic cancer surgery, the five-year overall survival rate for patients with pancreatic head cancer following curative resection remains disappointingly below 20% (49). The clinical efficacy of commonly used standardized chemotherapy drugs, such as gemcitabine, falls short of expectations. Additionally, drugs targeting pathways related to cell apoptosis for the treatment of pancreatic cancer are still in the developmental stage (50–52). The utilization of machine learning (ML) algorithms empowers comprehensive analysis of intricate and high-dimensional biological data encompassing genomics, transcriptomics, proteomics, and clinical records. This ML-driven approach enables the detection and elucidation of latent patterns, correlations, and non-linear associations that elude conventional statistical techniques. Leveraging this inherent capacity, ML facilitates the discernment of previously unexplored tumor biomarkers that hold significant clinical relevance and novelty (53, 54). The application of artificial neural networks for pancreatic cancer diagnosis has been scarcely explored in the existing literature. Furthermore, previous studies investigating serum miRNAs as potential biomarkers have generally exhibited limited specificity and sensitivity. Notably, the combination of circulating miRNAs with high sensitivity and specificity often requires the use of more than five miRNAs, a significantly larger number than what has been examined in our study. This observation suggests that the clinical implementation of such approaches may entail higher costs.

In this study, we conducted a comprehensive analysis of miRNA expression profiles of PCRSMs using two GEO datasets. Through the utilization of three machine learning algorithms, namely SVM-RFE, LASSO regression analysis, and random forest, we successfully identified three miRNA features. Subsequently, we developed a novel diagnostic model for PCRSMs based on an artificial neural network, which exhibited high sensitivity in detecting pancreatic cancer. To validate the performance of our model, we applied it to an independent GEO dataset and found that the ROC value was superior to that of the training set, indicating its robustness and generalizability. Moreover, we constructed a nomogram and calibration curve to further assess the accuracy of our diagnostic model. Additionally, by dividing the expression levels of PCRSMs into two different subtypes, we analyzed the correlation between PCRSMs and clinical characteristics. Our comprehensive analysis demonstrated that the diagnostic model built upon the artificial neural network outperformed similar models in terms of predictive performance. Furthermore, there was a strong agreement between the predicted values generated by our model and the measured values. The findings of this study provide physicians with a reliable framework for clinical decision-making, enhancing the ability to diagnose pancreatic cancer accurately and facilitating appropriate treatment strategies.

The present study investigated the utilization of three specific miRNA features, namely hsa-miR-4648, hsa-miR-125b-1-3p, and hsa-miR-3201. Hsa-miR-4648 has garnered significant attention as a potential biomarker for predicting recurrence in small cell carcinoma of the colon, cervix, and esophagus. Additionally, it has been implicated in neurodegenerative diseases such as ALS, highlighting its multifaceted role across various pathological contexts (55–58). Furthermore, hsa-miR-4648 has demonstrated a robust association with primary liver cancer susceptibility, as well as the risk of tumor progression and metastasis (59). Hsa-miR-125b-1-3p was identified as a valuable biomarker for the early diagnosis of numerous pancreatic cancers, underscoring its pivotal role in the development of pancreatic cancer. Moreover, its involvement in endothelial cell apoptosis and vascular injury provides novel insights into the pathogenesis of pancreatic cancer and holds potential implications for the development of innovative treatment strategies (60). Notably, our findings revealed a significant negative correlation between the expression of hsa-miR-125b-1-3p and age in pancreatic cancer patients, thus laying the foundation for personalized treatment plans tailored to individual patients. Hsa-miR-3201 has been extensively investigated in various malignancies, including hepatocellular carcinoma, recurrent epithelial ovarian cancer, melanoma, and pancreatic ductal carcinoma. It has been utilized for prognostic purposes and evaluating its expression levels in these diverse contexts, shedding light on its involvement in distinct cancer-promoting pathways (61–64). In summary, the inclusion of hsa-miR-4648, hsa-miR-125b-1-3p, and hsa-miR-3201 as miRNA features in our study showcases their significance in different cancer types and highlights their potential as biomarkers. This knowledge not only expands our understanding of their functional roles but also has implications for diagnostic and therapeutic strategies in the field of cancer research.

To address the inherent heterogeneity and diversity of pancreatic cancer, which can lead to significant variations in expression patterns and signaling pathways among samples, we employed a strategy to enhance sample size, improve clustering analysis accuracy and reliability, and minimize random errors resulting from data partitioning. Specifically, we combined two GEO datasets from the training set with one GEO dataset from the validation group (65). Through this integration, we successfully categorized pancreatic cancer into two distinct molecular subtypes, namely C1 and C2, based on the expression data of characteristic PCRSMs (66). The two molecular isoforms show significant differences. Notably, our clinical correlation studies revealed that all three PCRSMs exhibited associations with the age of pancreatic cancer patients. However, statistical significance was observed only for miR-125b-1-3p, while biological sex did not appear to be an influencing factor for the altered expression of the characteristic PCRSMs in pancreatic cancer patients. These findings provide a solid foundation for our future investigations, aimed at exploring personalized treatment plans for different types of pancreatic cancer patients, thus introducing novel perspectives and approaches in this field (67).

While our study has provided valuable insights into the early diagnosis of pancreatic cancer patients, it is important to acknowledge certain limitations. Firstly, the use of publicly available datasets of blood samples instead of qPCR experiments with serum samples from pancreatic cancer patients represents a limitation of our study. The limited availability of such samples in the hospital necessitated the use of cell lines as an alternative. This substitution may introduce biases into our results, which should be considered when interpreting the findings. In future investigations, we aim to overcome this limitation by collecting a larger number of serum samples from pancreatic cancer patients for qPCR validation. Furthermore, our study focused on the expression profiles of serum miRNAs in pancreatic cancer patients without investigating their functional roles. Therefore, the underlying mechanisms linking hsa-miR-4648, hsa-miR-125b-1-3p, hsa-miR-3201 with tumor immune infiltration and pancreatic cancer require further exploration. Future studies should aim to elucidate the functional significance of these miRNAs and uncover the specific pathways and interactions through which they contribute to pancreatic cancer progression. By addressing these limitations and conducting further investigations, we can advance our understanding of the role of serum miRNAs in pancreatic cancer and explore their potential as diagnostic biomarkers and therapeutic targets.

## Conclusion

Our study presents a novel artificial neural network (ANN) model with promising clinical implications for the early detection of pancreatic cancer. This ANN model demonstrates exceptional performance in accurately distinguishing pancreatic cancer samples from normal samples and effectively predicting the characteristics of previously unobserved samples. Leveraging comprehensive bioinformatics analysis, we extensively investigate the expression profiles of pancreatic cancer-specific miRNAs (PCSMs) and elucidate their associations with clinical traits. Our findings unveil significant correlations between specific PCSMs and patient age, thereby highlighting their potential relevance in drug screening, personalized treatment approaches, and immunotherapy for pancreatic cancer. These discoveries offer fresh insights and lay the groundwork for future investigations in the realm of pancreatic cancer management.

## Data availability statement

The original contributions presented in the study are included in the article/**Supplementary Material**. Further inquiries can be directed to the corresponding authors.

## Author contributions

YL, QZ and GY conceived the study. HQC, HC, RW, LJ, SZ, CJ, JZ and XQ drafted the manuscript. HQC, JH, YL and QZ performed the literature search and collected the data. HQC and HC analyzed and visualized the data. HC and RW completed cellular experiments. HC, QZ and GY helped with the final revision of this manuscript. All authors contributed to the article and approved the submitted version.

## Supplementary material

Supplementary Figure 1 Verification of hsa-miR-4648 by qRT-PCR. The expression of hsa-miR-4648 was significantly upregulated in PC tissues, deviating from the expected results.
