# Supplementary figures and images for: Proposing new early detection indicators for pancreatic cancer: Combining machine learning and neural networks for serum miRNA-based diagnostic model
Source: Front Oncol. 2023 Aug 3;13:1244578. doi: 10.3389/fonc.2023.1244578 (PMC10437932; doi:10.3389/fonc.2023.1244578)

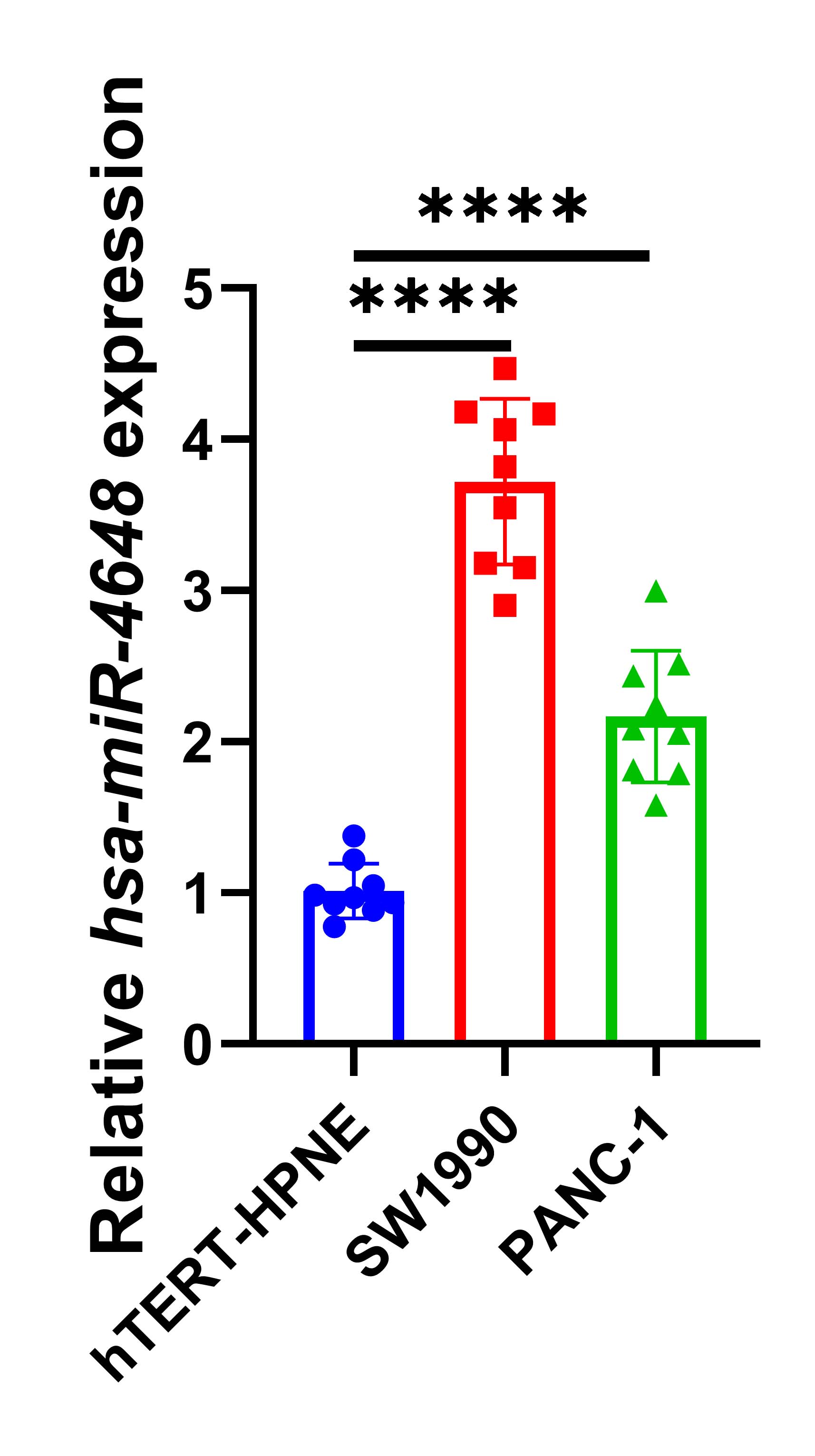

Supplement: Supplementary Figure 1 — Verification of hsa-miR-4648 by qRT-PCR. The expression of hsa-miR-4648 was significantly upregulated in PC tissues, deviating from the expected results. [file Image_1.jpeg]
